# Sustainable development, eco-tourism carrying capacity and fuzzy algorithm-a study on Kanas in Belt and Road

**DOI:** 10.1038/s41598-023-41961-1

**Published:** 2023-10-05

**Authors:** Kui Yu, Han Gao

**Affiliations:** 1 Glorious Sun School of Business and Management, Donghua University, Shanghai, China; 2 College of Fashion and Art Design, Donghua University, Shanghai, China

**Keywords:** Ecology, Environmental social sciences

## Abstract

In this paper, the method of fuzzy pattern recognition is adopted in more precisely evaluating the actual state of eco-tourism development regarding a given tourist destination in comparison with the three standard patterns (saturated/optimal/deficient) of development degree. The research process is as follows: Firstly, the indictors of carrying capacity of a tourist destination and the corresponding measuring factors are established; secondly, an assessment group is recruited to work out the most constraining factors among the measuring factors; thirdly, by means of field survey, numerical values of the actual state are acquired; fourthly, there comes out the membership vectors and the membership matrix of the standard patterns corresponding to the vectors of three standard patterns, threshold and the actual state; Finally, it could be identified which standard pattern that the actual state is closest to via the lattice degrees of proximity. An exemplary case study on Kanas National Nature Reserves is attached to the logic calculus. This paper is contributed to dynamically monitor the threshold of tourism carrying capacity and precisely identify which carrying capacity (spatial resource/ecological environment/economic resources/people’s psychology/socio-culture) with potential risks.

## Introduction

The ideology of sustainable tourism development was formed in the early 1990s. The main frameworks and objectives of sustainable tourism development theory were elaborated by the Globe’90 International Conference held in Canada in 1990 as: (1) Strengthening ecological awareness; (2) Promoting equity development; (3) Improving quality life of local community; (4) Providing high-quality experience to the tourists; (5) Protecting the environment which tourism development relies on in the future. The main frameworks and objectives are aimed to coordinate in the interests of environment, tourists and local community^1^.

According to Pigram, sustainable tourism has the potential to be a tangible expression of sustainable development. Its core issues are intra-generational equity and inter-generational equity in the future, as well as a series of social equity rules (i.e. equity between the output of tourist attractions and the costs of protecting the environment)^2^. The eco-tourism has been growing rapidly in recent years, in particular with the rapid spread of social media (i.e. Instagram, or Little Red Book in China), these new forms of tourism in popularity are especially apt to mutate into mass tourism (i.e. increasing number of tourists, intensive marketing, large accommodations and transportations in need, changes in products, and the impact on the destination). Tourism, like any other industries, would inevitably damage the environment in the long run. Moreover, due to the cancer-like nature of tourism exploitation, it tends to invade more remote, undamaged areas accompanied by more established tourist attractions being overdeveloped^3,4^. New airstrips and hotels are being built at a growing rate, delivering mass tourism to the most remote places and islands. Tourism can therefore be a contributing variable in the environmental degradation throughout regions and countries. Generally, big cities seem to be more resilient to the tourist invasions than the wide open countryside and a variety of particularly fragile environments (i.e. islands, coral reefs and oases). The impact on small towns would be more severe than on big cities, due to being less ecologically and culturally resilient to the tourist invasions.

The challenges facing the tourism are even harder under the pandemic. After the lockdown of the first half of 2022, many people were desperately getting out. Many places were gearing up for a wave of retaliatory consumption. Along with the summer holidays coming, a vengeful tourism boom indeed arrived in Sanya, a landmark of travel destinations, was swept by the epidemic, leaving more than 80,000 tourists retained on the island in July, 2022. Starting from January, 2023, passport applications have been resumed for outbound travelers in China. This is coupled with the removal of COVID tests and quarantine for all inbound traffic. The global tourism industry, which has been in the downturn for the last three years, is likely about to recover in a highly stressful way. Statistics show that in the first five months of 2022, global tourism strongly rebounded with about 250 million global tourists, double the number of 2021. In some regions, arrivals have already reached, or even exceeded the pre-pandemic levels. Organization for Economic Co-operation and Development (OECD) pointed out that “Governments need to rethink their tourism policies to encourage more diversity, reducing concentration in high-density destinations and putting in place long-term strategies that are ecologically sustainable and socially inclusive.” The theme of World Tourism Day on September 27, 2022 is exactly “Rethinking Tourism”. Tourism has taken on a new meaning in the wake of the global fight against COVID-19. People come into thinking deeply about tourism, which means that humanity should get along with the planet in a more united and environmentally friendly way.

After this wave of outbreaks in tourist destinations, people can’t help reflecting on how to dynamically and scientifically monitor the carrying capacity of tourist destination in condition of social distance required by the epidemic prevention. How to brake in time before the irreversible impacts are caused in excess of the threshold? How to predict the eco-tourism would be turned into a mass tourism? How to measure the ultimate capacity of exploitation and usage of a tourist attraction? Conducting scientific research and assessment on the state of eco-tourism environmental carrying capacity in the manner of “threshold” may be the key to the problem.

This paper is pragmatically trying to respond the above questions via indirect method of patter recognition with application of fuzzy algorithm. The exploitation and usage state of a given eco-attraction can be scientifically discriminated by measuring the carrying capacity. The skill is in help with dynamic monitoring and risk pre-warning on the imminently breakable carrying capacity for the management department, coping with the seasonal crowd surging by all accounts. Firstly, the literatures are sorted out in this paper consistent with the relation between sustainable development, sustainable tourism and carrying capacity; secondly, the indicators of eco-tourism environmental carrying capacity are established; thirdly, in application of fuzzy pattern recognition, the exploitation and usage state of eco-tourism carrying capacity is dynamically discriminated; fourthly, the indirect method of fuzzy pattern recognition is applicable to an exemplary case of the hit tourist attraction Kanas in Xinjiang Uygur Autonomous Region (abbreviated as “Xinjiang”) China, aiming to get hold of “threshold” related to Kanas and scientifically control the risk of excessive development.

## Reference review

### Sustainable development

The term sustainable development in evolution is generally supposedly initiated by the increased environmental awareness in the 1960s and 1970s^5–10^. In response to the failure of the economic growth model during Post-World War II, it was called for an alternative, more sustainable model of development.

Early ideology of sustainable development was proposed at the international conferences (Stockholm Conference on People and the Environment, 1972) and the conceptualisations in literatures as well as conferences in form of The Limits to Growth^11^, Ecological Principles of Economic Development^12^, the Brandt Commission Report 1980, and Our Common Future (World Commission on Environment and Development (WCED), 1987). Sustainable development was subsequently in discussion at the G7 Summit in Paris in 1989^13^, World Conservation Strategy in 1981 [International Union for Conservation of Nature and Natural Resources (IUCN)], United Nations Environment Programme (UNEP), World Wide Fund for Nature (WWF 1991). Sustainable development in Our Common Future was defined as a “process to meet the needs of the present without compromising the ability of future generations to meet their own needs” (WCED 1987). Sustainability is possessed of three fundamental elements (ecological, socio-cultural, economic), and three fundamental principles (future, equity, holism)^14^.

### Sustainability in tourism and sustainable development

The profound and rapid changes that have been taking place in the world over the past two decades are embodied in the changes in tourism. In comparison with the expansion of tourism scale, the introduction of “sustainability” is the exercisable variable most likely to change the nature of tourism. Rosenow and Pulsipher called for “new tourism” that does not exceed carrying capacity, protecting towns, enhancing environmental and heritage values, and educating visitors^15^. Butler’s life cycle theory of tourist destinations^16^ was considered indirectly reflecting the concept of sustainable development^9^ and the concept of carrying capacity^17,18^.

Since the publication of Brundtland Commission Report Our Common Future (WCED 1987) in 1987, the term sustainability has been shifted from the ideology of sustainable development into the tourism industry. According to current literatures, sustainable tourism is generally defined as,

tourism which is developed and maintained in an area (community, environment) in such a manner and at such a scale that it remains viable over an infinite period and does not degrade or alter the environment (human and physical) in which it exists to such a degree that it prohibits the successful development and well-being of other activities and processes^19^*.*

**Figure 1:**
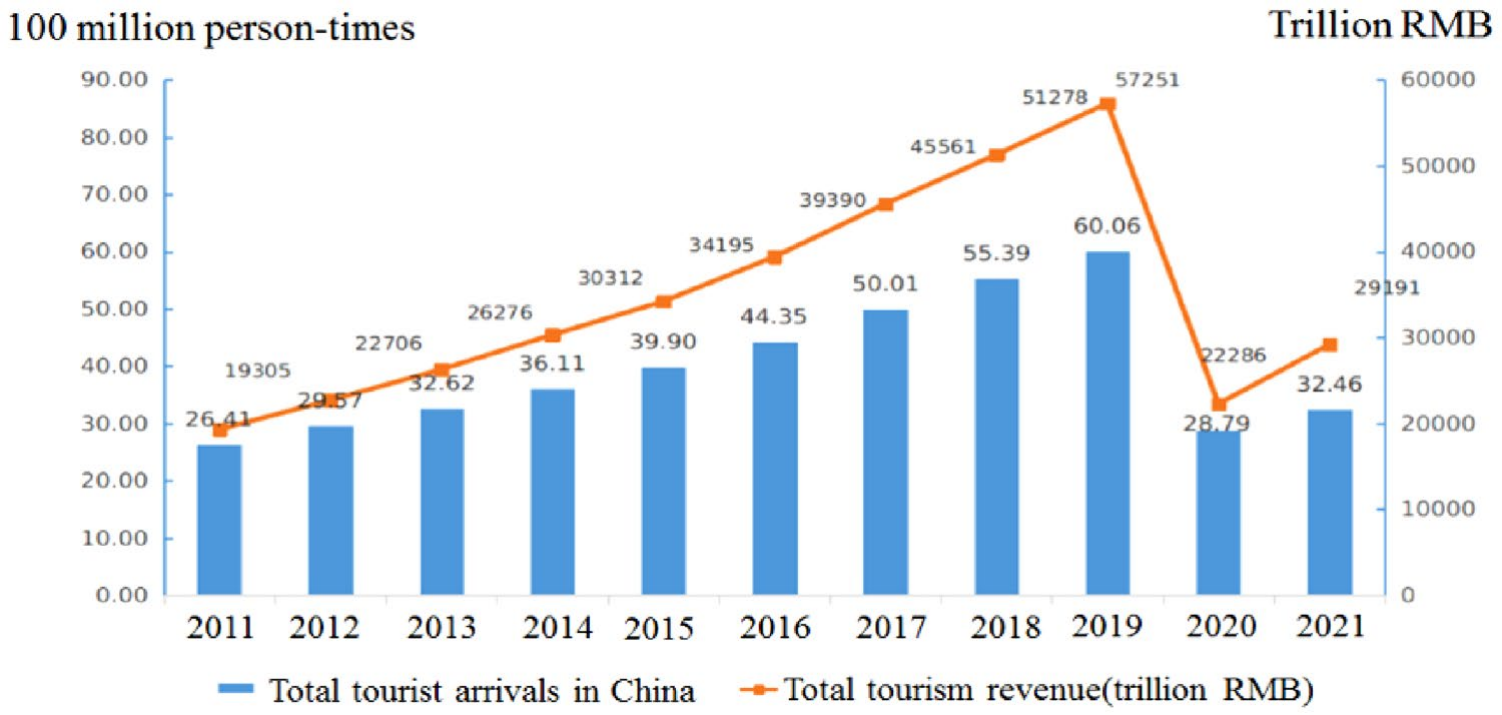
The tourism in China has consecutively been undergoing rapid growth from 2011 to 2019. Tourism revenue resolutely surged to an all-time high of 5.725 billion RMB in 2019. Even with the devastating impact of covid-19 in 2021, the total number of Chinese domestic tourists reached 3.246 billion, the domestic tourism revenue (total tourism consumption) mount to as much as 2.92 trillion RMB^20^.

Tourism is a key sector to the national economic development and social well-being of people. Take China as an example (Fig. 1).

Therefore, inserting the word “tourism” in the middle of “sustainable development” is an effort to converge the two lines into one and transform this concept into action. Pigram also expressed a similar point of view^2^.

Sustainable tourism has the potential to become a tangible expression of sustainable…development. Yet it runs the risk of remaining irrelevant and inert as a feasible policy option for the real world of tourism development, without the development of effective means of translating the idea into action.

The term “sustainable tourism” is now widely used in the tourism literatures. However, the definition, validity and operability of the term have been in controversy all the time. As far as the definition is concerned, sustainable tourism is criticized for being narrow and sectoral^9–11,19^. Sustainable tourism may share some concerns with sustainable development. However, sustainable tourism has its own specific tourism-centric agenda in the aim of maintaining business viability, sometimes being understood as an ideology and perspective rather than an explicitly operational definition^21^. Some organizations, such as the WTO Task Force, choose not to particularly define “sustainable tourism”. They propose that it is a concept applicable to a specific place or destination and should therefore be defined on a case-by-case basis^22^.

Cater believes that there are four loose stakeholders (host community, tourist, tourism operators and regulators, natural environment), which respectively play an equally important role in developing natural environment and managing tourist products in executing sustainable tourism development^23^. The tourism system should be built in the interests of all stakeholders. It is asserted by Muller that the objectives of sustainable tourism are in essence comprised of the following factors^24^,

- economic health,
- subjective well-being of the locals,
- unspoilt nature, protection of resources,
- healthy culture and
- optimum satisfaction of guest requirements.

Muller depicts the objectives of sustainable tourism as a balanced tourism development, no one dominating^24^. In fact, Hunter regards sustainable tourism as^25^, need not (indeed should not) imply that these often competing aspects are somehow to be balanced. In reality, trade-off decisions taken on a day to day basis will almost certainly produce priorities which emerge to skew the destination area based tourism environment system in favor or certain aspects.

Moreover, the opinions of Healey and Shaw are phrased as^26^, preference for the conception of balances and trade-offs not only sits more comfortably with economic priorities, it is also more easily subverted by imperatives of economic growth in that environmental limits to a trade-off are not set.

Over the past decades, sustainable tourism has been in dispute over how to put it into practice. The solutions to this issue are running through the literatures related to sustainable development. Sara et al. analyzed the topics of 20 articles most cited during 2019–2020 and concluded that the contribution of tourism to economic growth and the measurement of sustainability are receiving more attention^27^. There is a voice that sustainable tourism is about reducing the use of the non-renewable resources. In light of the carrying capacity, sustainable products or minimum safety standards^2^, researchers can’t help wondering to what extent should a precautionary development put into effect averse to the risks of exceeding the limits of carrying capacity^28^? Under this background, carrying capacity is put forward as a paradigm to ascertain and constrain the usage of a tourist destination^29–32^. Nowadays, tourism carrying capacity, as a solution on local scale, has been widely adopted in tourism research, aiming to provide more specific, time**/**space solutions at the local level.

### The main measuring methods of tourism carrying capacity

The concept of carrying capacity was initiated in the field of rangeland and wildland management. Four types of classification are as follows: physical carrying capacity, perceptual carrying capacity, social carrying capacity and economic carrying capacity, which are the methodological basis for measuring carrying capacity^33^. The logistic model was combined with carrying capacity by Odum, but it is pointed out that the logistic model is a deterministic model solely applicable to the laboratory situation, in which the world is presumptively a deterministic closed system rather than an open system. Therefore, the application of logistic model in carrying capacity is prone to be a misleading. One of the criticisms of carrying capacity is the abstract nature and the inconsistency of the measured values with the realities of a particular area^34,35^. Lack of a comprehensive definition of carrying capacity, the ever-changing nature of the concept and the variety of approaches to measurement have accounted for the criticism. The interface to all methods of measuring carrying capacity is the identification and determination of the limit of acceptable variation (LAC), which is the alternative basis of measurement. Various approaches, such as weighted valuation, multi-criteria ranking of capacity, management models adapted to ecosystems, ecological footprint models, and other simple and composite models have been used to measure physical, ecological, social and perceptual carrying capacity^36,37^. The guideline put forward by International Union for Conservation of Nature (IUCN) in 1996 applied to computing the carrying capacity of regions are suitable for tourism development within the protected zones^22^. Since the twenty-first century, scholars began to pay more attention to the quantitative research and practice of tourism environmental carrying capacity. Saveriades established a mathematical model to conduct an empirical study on the tourism social carrying capacity of Cyprus parks^37^. Tony Prato proposed the Adaptive Ecosystem Management (AEM) and Multiple Attribute Scoring Test of Capacity (MAS⁃TEC) models, which were adopted to assess the carrying capacity of national parks in the United States^38^. Steven Lawson et al.^39^ expanded and elaborated on the use of computer simulation modeling as a tool for proactive monitoring and adaptive management of social carrying capacity at Arches National Park^35,36^.

The thresholds have been measured in many case studies^40^. Shelby and Heberlein argued that carrying capacity should be determined by studying tourist expectations as well as predetermined rules of destination managers. The two sociologists expanded the conceptual basis by differentiating the types of activities that tourists engage in natural areas, establishing a model of social carrying capacity that is still valid today and has been applied to natural areas^41^. Vaske and Shelby reviewed the studies on perceived crowding between 1975 and 2005 (all at recreational venues)^42^. It is concluded that the criteria proposed by Shelby and Heberlein^43^ remain a viable methodology for assessing carrying capacity based on perceived crowding levels. Seidl and Tisdell^38^ outlined that the considerable uncertainty to the measurement of carrying capacity are attributed to the highly variable nature of the environment, the nonlinear dynamic nature of many causal relationships and the lack of knowledge. Saveriades stated that carrying capacity is more of a dynamic and fluid concept^44^. In addition to measurement issues, the impact of carrying capacity varies in line with the destination and also depends on destination management processes^45^. Some scholars believe that establishing the purpose of a region is crucial for determining the carrying capacity. Therefore, for any region, there are various carrying capacities, implying no single capacity can apply to the whole region^46^. Simon et al. conducted qualitative research and highlighted the major problems in measuring carrying capacity in manner of different ways to reach a destination and no specific standard way to measure carrying capacity^39^. Few studies have discussed the problems in measuring the carrying capacity of tourist destinations under pressure. Although significant progress has been made in evaluating carrying capacity, most current methods are non-quantitative and lack analytical rigor.

The studies on environmental carrying capacity in China are mainly in the aspects of the minimum quantity law, namely the barrel theory, and the weight of measurement and evaluation indictors in application of mathematical model. Cheng Zhen et al. proposed the law of minimum tourism environmental capacity and built a mathematical model^47^. Cui Fengjun et al. proposed a static model for measurement in result of exploring the connotation and composition of tourism environmental carrying capacity. Given by social, cultural, economic, psychological and other factors of tourist destinations, a systematic tourism carrying capacity indicator was brought up^48^. Liu Mei et al. adopted Pressure-State-Response (PSR) Model to construct the evaluation model of eco-tourism environmental carrying capacity of water eco-tourism scenic spots^49^. The conceptual system and evaluation of tourism environmental carrying capacity in China are in terms of single-factor measurement, and there is insufficient research on the comprehensive evaluation model involving multiple factors.

In conclusion, it is identified that those works aimed at providing indicators or systems for measuring sustainability are limited to a specific region. In addition, the lack of data makes the accuracy of the studies difficult. It is essential to expand empirical studies in this field^50^. Taking the eco-tourism environmental carrying capacity as a paradigm for the study of sustainable tourism, with application of fuzzy algorithm, this paper is empirically dedicated to assessing the actual state of eco-tourism development degree (excessive**/**moderate**/**insufficient), as a reference for risk evaluation and scheme adjustment. The study procedure is as following: (1) Establishing the indicator of eco-tourism environmental carrying capacity and corresponding measuring factors, followed by building standard patterns; (2) On account of fuzzy algorithm, adopting fuzzy pattern recognition to assess the actual state of eco-tourism environmental carrying capacity, identifying to which standard pattern it is most approximate to. The tourism planners and local organizations can take this method as a powerful and useful tool to dynamically monitor the state of carrying capacity. As a result, the potential exploitation capacity, resilience, integrity of structure and function regarding the natural resources, as well as the long-term well-being of ecosystem can be guaranteed.

## The mathematical expression of fuzzy pattern recognition

**Figure 2:**
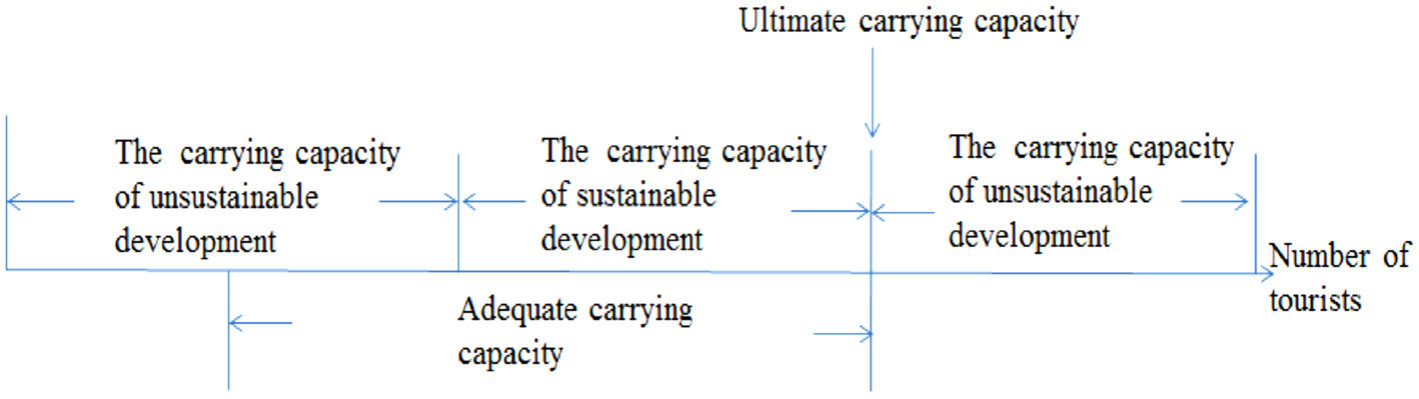
There are two main aspects integrated into the definitions of tourism carrying capacity: (1) the biophysical component, connecting to the integrity of the resource-base which suggests some specific threshold or level of tolerance after which further exploitation or usage may put pressure on the natural ecosystem; and (2) the behavioral component, in response to the quality of tourism experience^17^.

In expression with a relative state, not a specific number, carrying capacity would be seemingly more consistent with the spirit and purpose of regulations. As long as the impact remains acceptable, the current usage of a tourist attraction could be continuously carried out. If the state becomes unacceptable and all other management strategies are unsuitable other than reducing the usage, the carrying capacity so far could be adjusted to the current usage to meet the requirements of regulations (Fig. 2).

Carrying capacity is defined by WTO as “the maximum number of people that can visit a tourist destination at the same time without causing disruption by physical, economic or socio-cultural means and an unacceptable reduction in tourist satisfaction”. Therefore, carrying capacity intrinsically indicates a curvilinear relationship between usage and impact, which would vary in capacity under different environmental and social conditions^51^. Tourism carrying capacity is considered to be a manipulative variable in the consecutively changing process. Consequently, the way of measuring carrying capacity should be suitable for the multi-objective, conditional, nonlinear and highly dynamic state of system^52^. As a matter of fact, the vaguely, imprecisely defined categories of scenarios, mostly encountered in the real world, are different from the ones explicitly defined in the traditionally mathematical manner due to the fuzziness featuring the boundaries of the categories. The so-called fuzziness goes by the name of fuzzy set, which means there is no sharp transform from membership to non-membership in a category of scenario. The difference between fuzzy algorithm and probability statistics is that the latter lacks the competence in grasping the fuzzy problem and dealing with higher-order complex systems^53^.

**Figure 3:**
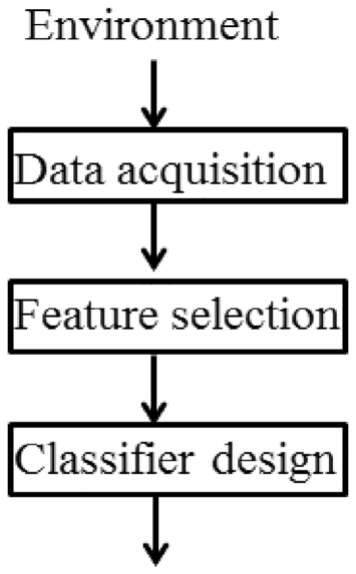
A general scheme of pattern recognition^55^.

A pattern is an ideally imitable sample, in form of fuzzy set. Pattern recognition refers to identifying which standard pattern an object is closest to. Sergios claims that Pattern recognition is a scientific discipline whose aim is the classification of the objects into a lot of categories or classes. Pattern recognition is also an integral part in most machine intelligence system built for decision making^54^. Pattern recognition is generally visualized as a sequence of some steps in process, namely (i) data acquisition, (ii) feature selection, and (iii) classification procedure (Fig. 3)^55^.

Fuzzy pattern recognition is based on fuzzy algorithm^51^. The Pattern recognition system based on fuzzy sets theory can imitate thinking process of human being widely and deeply^56–59^. The purpose of this paper is about to evaluate the actual state of a given tourist destination, and to identify its development degree (excessive**/**moderate**/**insufficient) by measuring which standard state it is closest to. The moderate development degree, acting as a transition between the excessive and insufficient development degree, is the ideal goal for a tourist destination. This paper is grounded in the fuzzy pattern recognition which is mathematically expressed as follows.

Assuming $A{ = }\left( {a_{1} ,a_{2} , \ldots ,a_{p} } \right)$ and $B{ = }\left( {b_{1} ,b_{2} , \ldots ,b_{p} } \right)$ being two *p*-dimensional fuzzy vectors (fuzzy set in expression with vectors), the symbol “$\wedge$”and “$\vee$”are respectively representing taking “minimum operation” and “maximize operation”.

The inner product $A \cdot B$ of the fuzzy vectors *A* and *B* is defined as

$$ A \cdot B{ = }\mathop \vee \limits_{{k{ = 1}}}^{p} \left( {a_{k} \wedge b_{k} } \right) $$

The outer product $A \odot B$ of the fuzzy vectors *A* and *B* is defined as

$$ A \odot B{ = }\mathop \wedge \limits_{k = 1}^{p} \left( {a_{k} \vee b_{k} } \right) $$

The lattice degree of proximity $N\left( { \, A{, }B \, } \right)$ between the fuzzy vectors *A* and *B* is defined as

1 $$ N\left( { \, A{, }B \, } \right) = \frac{1}{2}\left[ {A \cdot B + \left( {1 - A \odot B} \right)} \right] $$

It is deduced from the definition that the closer the lattice degree of proximity regarding two fuzzy vectors gets to 1, the more approximate the two fuzzy vectors are. On the contrary, the closer the lattice degree of proximity regarding two fuzzy vectors gets to 0, the estranged the two vectors are.

Assuming *A* and *B*_1_, *B*_2_, …, *B*_*n*_ are both *p*-dimensional fuzzy vectors, in existence of $j_{0} \in \left\{ {1,2, \cdots \cdots ,n} \right\}$, on the nearest neighbor rule, the following formula is on hand:

2 $$ N\left( {A{, }B_{{j_{0} }} } \right) = \max \left\{ {\begin{array}{*{20}c} {N\left( { \, A{, }B_{1} } \right),} & {N\left( {A{, }B_{2} } \right),} & { \ldots ,} & {N\left( {A{, }B_{n} } \right)} \\ \end{array} } \right\} $$

from which *A* would be inferentially categorized into pattern $B_{{j_{0} }}$ under conditions that *A* is closest to $B_{{j_{0} }}$.

## Indicators of eco-tourism environmental carrying capacity

Carrying capacity, in connection with the evaluation criteria reflecting an objective or an expected state, assures the tourism development of marching in the right direction. The indicators of sustainable tourism should be endowed with relevance, resonance, reliability and simplicity, accompanied by measuring factors which are carefully selected and properly screened.

This study constitutes the indicator of eco-tourism environmental carrying capacity, consistent with the research by Jinmei and Jian^60^, containing five componential indicators as carrying capacity of spatial resource (*C*_1_), carrying capacity of ecological environment (*C*_2_), carrying capacity of economic environment (*C*_3_), carrying capacity of people’s psychology (*C*_4_) and carrying capacity of socio-culture (*C*_5_). The measuring factors affiliated with each indicator are shown in Table 1.

**Table 1:** Indictors of eco-tourism environmental carrying capacity.

Sequence	Indicators of carrying capability	Denoted as	Measuring factors
1	Spatial resource carrying capacity^48^	*C*_1_	Touring routes capacity (width, length and traits, etc.)
			Areas capacity (types, geology and geomorphology of tourist attractions in the reserves, etc.)
			Ecological capacity (regional environment and scenic spots in the reserves)
			Crossroads capacity (the ways for visiting, etc.)
2	Ecological environment carrying capacity^9^	*C*_2_	Water environmental pollution indicators (standards for chemical and biochemical oxygen, amounts of bacteria, etc.)
			Air pollution indicators (concentrations of PM_2.5_, PM_10_, SO_2_, NO_2_, CO, O_3_, etc.) Solid waste disposal indicators (environmental purification amount of pollutants, purification time, etc.)
3	Economic resources carrying capacity^48^	*C*_3_	Infrastructural capacity (traffic and hydropower supply, etc.)
			Tourist reception capacity (accommodation, entertainment, etc.)
4	People’s psychological carrying capacity^9^	*C*_4_	Residents’ psychological capacity (average number of tourists tolerated by residents in tourist destinations, etc.)
			Tourists’ psychological capacity (density of crowding tourists are not resentful, etc.)
5	Socio-cultural carrying capacity^9^	*C*_5_	Impact on cultural facilities, lifestyles and customs (protection of cultural relics and respect for local lives in tourist destinations, etc.)
			Frequency of cultural exchanges held in a year

From the measuring factors associated with the five indicators of carrying capacity (Table 1), it can be seen that some are hard indicators for quantitative analysis, while some are soft indicators for qualitative analysis. The degrees to which a tourist attraction is being developed and used, are categorized into three fuzzy standard patterns (excessive**/**moderate**/**insufficient), objectively reflecting the degree of adequateness for development. For this purpose, the investment developers, tourism authorities, local governments, experienced experts and local communities of a tourist destination (referred to as “assessment group”) are recruited for periodically assessing the eco-tourism environmental carrying capacity of a tourist destination. Firstly, the decisive factor, among the measuring factors of each carrying capacity *C*_*i*_(*i* = 1,2,…,5), extremely constraining the tourism development, is the referral factor easiest to reach saturated state. The constraining factors are to be figured out among the measuring factors, which is consistent with the barrel theory (the poorly done and most disadvantaged part determine the development of an organization). Given by the constraining factors, as well as the actual experiential data in recent years, statistical data, medium and long-term development planning for the tourist destination, the roughly numerical values of saturated/optimal/deficient standard patterns expressed as *c*_*i*1_**/***c*_*i*2_**/***c*_*i*3_ (take the mid-value if only an interval range determined) are resulted. Thus, the vectors of the three standard patterns of eco-tourism environmental carrying capacity are generated in formula (3):

3 $$ \left\{ \begin{gathered} S_{1} = \left( {\begin{array}{*{20}c} {c_{11} } & {c_{21} } & \cdots & {c_{51} } \\ \end{array} } \right) \hfill \\ S_{2} = \left( {\begin{array}{*{20}c} {c_{12} } & {c_{22} } & \cdots & {c_{52} } \\ \end{array} } \right) \hfill \\ S_{3} = \left( {\begin{array}{*{20}c} {c_{13} } & {c_{23} } & \cdots & {c_{53} } \\ \end{array} } \right) \hfill \\ \end{gathered} \right. $$

Hereafter *S*_1_**/***S*_2_**/***S*_3_ are respectively named after the vectors of saturated**/**optimal**/**deficient standard patterns, referral to the excessive**/**moderate**/**insufficient development degrees to the tourist destination. Taking the three vectors as column vectors, the standard pattern matrix of the eco-tourism environmental carrying capacity accrues as follows:

4 $$ C = \left( {\begin{array}{*{20}c} {\begin{array}{*{20}c} {c_{11} } \\ {c_{21} } \\ \vdots \\ {c_{51} } \\ \end{array} } & {\begin{array}{*{20}c} {c_{12} } \\ {c_{22} } \\ \vdots \\ {c_{52} } \\ \end{array} } & {\begin{array}{*{20}c} {c_{13} } \\ {c_{23} } \\ \vdots \\ {c_{53} } \\ \end{array} } \\ \end{array} } \right) = \left( {c_{ij} } \right)_{5 \times 3} $$

of which *c*_*ij*_ represents the numerical value of standard pattern *j* pertinent to the carrying capacity *i* (*i* = 1, 2, 3, 4, 5; *j* = 1, 2, 3). It should be noticed that the validation of *c*_*ij*_, the basis for evaluating the development and usage state of tourist attraction, is vital. For example, it is roughly verified by the assessment group that the area capacity, a hard indictor for quantitative analysis in nature, is the decisively constraining factor among the measuring factors (touring routes capacity, area capacity, ecological capacity, crossroads capacity), with respect to the spatial resource carrying capacity *C*_1_ regarding a given tourist destination. The following formula stands out correspondingly:

$$ c_{1j} = \frac{{D_{j} }}{d} \times \frac{{T_{j} }}{t} $$

Here *D*_*j*_**/***d***/***T*_*j*_**/***t* are respectively designated as tourist areas**/**per person occupied areas**/**opening hours (could be day**/**week**/**month**/**year)**/**the time for sightseeing. The formula in expression of *c*_1*j*_, which is treated as the number of people (including tourists and working staff) accommodated by the tourist destination, is capable of reasonably measuring the carrying capacity of spatial resources. In response to the determination of *D*_1_**/***D*_2_**/***D*_3_ and *T*_1_**/***T*_2_**/***T*_3_ by the assessment group, the large**/**appropriate**/**low receiving capacity, in name of *c*_11_**/***c*_12_**/***c*_13_, referring to the numerical values of saturated**/**optimal**/**deficient standard patterns in regard to the tourist destination, hence is got touched. To give another case, the residents’ psychological capacity, the most constraining factor among the measuring factors (residents**/**tourists’ psychological capacity) subject to the people’s psychological carrying capacity *C*_4_, is roughly verified by the assessment group. Therefore arises the numerical values of saturated**/**optimal**/**deficient standard patterns in name of *c*_41_**/***c*_42_**/***c*_43_, as a matter of fact, corresponding to the averaging of the number of visitors the local residents could tolerate resulted from questionnaire and**/**or field survey.

The eco-tourism environmental carrying capacity is verifiably capable of being self-regulated, resilient and self-preserving to the ecosystem of a tourist destination. Therefore, eco-tourism environmental carrying capacity usually exhibits in form of “threshold”, as much as to say that exceeding the “threshold” would most likely compromise the functional play and maintenance of the ecological environment system. Accordingly, in addition to locate the numerical value of *c*_i_, the vector of threshold in form of $$S_{\max } = \left( {\begin{array}{*{20}c} {c_{1\max } } & {c_{2\max } } & \ldots & {c_{5\max } } \\ \end{array} } \right)$$ should be taken into consideration. The threshold is written as *c*_*i*max_ (*i* = 1, 2, 3, 4, 5), corresponding to the maximum limit of each carrying capacity, which should be met with the following formula (5) for each *i*:

5 $$ c_{i\max } - c_{i2} \ge \left| {c_{ij} - c_{i2} } \right|\quad \left( {j = 1,2,3} \right) $$

Back to the first case above, the threshold *c*_1max_ of the spatial resource carrying capacity *C*_1_ generally indicates the maximum receiving capacity of a given tourist destination.

## The state evaluation matrix in application of fuzzy pattern recognition

Since the numerical values of each standard pattern vary in magnitude and dimension, lacking in comparability, the standard pattern matrix *C* in formula (4) should be correspondingly transformed into the membership matrix in application of fuzzy algorithm. Considering that the “nearest neighbor” rule is met for any numerical value, the following formula (6) is adopted for normalization process:

6 $$ c_{ij}^{*} = 1 - \frac{{\left| {c_{ij} - c_{i2} } \right|}}{{c_{i\max } - c_{i2} }}\quad \left( {i = 1,2,3,4,5;\,j = 1,2,3} \right) $$

It can be told from formula (5) that the numerical value *c*_ij_^*^ is located between closed interval [0, 1], namely $0 \le c_{ij}^{*} \le 1$. It turns out the numerical values of all the optimal standard patterns are transformed into 1, denoted as $c_{i2}^{*} = 1\quad \left( {i = 1,2,3,4,5} \right)$, obviously highlighting “the more moderate the better” rule. *C*_ij_^*^ is named after the degree to which the standard pattern *j* of the carrying capacity *i* is subordinate to the optimal standard pattern. Under this circumstance, the optimal tourism output accrued from the componential carrying capacities is released, followed by the membership vectors (formula 7) corresponding to the vectors of the three standard patterns of eco-tourism environmental carrying capacity:

7 $$ \left\{ \begin{gathered} S_{1}^{*} = \left( {\begin{array}{*{20}c} {c_{11}^{*} } & {c_{21}^{*} } & \cdots & {c_{51}^{*} } \\ \end{array} } \right) \hfill \\ S_{2}^{*} = \left( {\begin{array}{*{20}c} {c_{12}^{*} } & {c_{22}^{*} } & \cdots & {c_{52}^{*} } \\ \end{array} } \right) \hfill \\ S_{3}^{*} = \left( {\begin{array}{*{20}c} {c_{13}^{*} } & {c_{23}^{*} } & \cdots & {c_{33}^{*} } \\ \end{array} } \right) \hfill \\ \end{gathered} \right. $$

which is respectively named after the membership vectors of saturated**/**optimal**/**deficient standard pattern, collectively called membership vectors of the three standard patterns. Apparently, the three vectors are fuzzy vectors.

Taking the three membership vectors as column vectors, hereafter the membership matrix C^*^ of the standard pattern matrix *C* is drawn out:

8 $$ C^{*} = \left( {\begin{array}{*{20}c} {\begin{array}{*{20}c} {c_{11}^{*} } \\ {c_{21}^{*} } \\ \vdots \\ {c_{51}^{*} } \\ \end{array} } & {\begin{array}{*{20}c} {c_{12}^{*} } \\ {c_{22}^{*} } \\ \vdots \\ {c_{52}^{*} } \\ \end{array} } & {\begin{array}{*{20}c} {c_{13}^{*} } \\ {c_{23}^{*} } \\ \vdots \\ {c_{53}^{*} } \\ \end{array} } \\ \end{array} } \right) = \left( {c_{ij}^{*} } \right)_{5 \times 3} $$

In pursuit of the standard pattern to which the actual state of the eco-tourism environmental carrying capacity is closest, the field survey is carried out. The actual state vectors of the carrying capacity as following is originated from the numerical values denoted as *c*_10_, c_20_, *c*_30_, *c*_40_, *c*_50_:

$$ S_{0} = \left( {\begin{array}{*{20}c} {c_{10} } & {c_{20} } & \ldots & {c_{50} } \\ \end{array} } \right) $$

Which are input into formula (6) for normalization, the result coming out:

$$ c_{i0}^{*} = 1 - \frac{{\left| {c_{i0} - c_{i2} } \right|}}{{c_{i\max } - c_{i2} }}\quad \left( {i = 1,2,3,4,5} \right) $$

The membership vector *S*_0_^*^ of the actual state regarding the carrying capacity *S*_0_ is as following:

$$ S_{0}^{*} = \left( {\begin{array}{*{20}c} {c_{10}^{*} } & {c_{20}^{*} } & \ldots & {c_{50}^{*} } \\ \end{array} } \right) $$

Apparently, the above is fuzzy vector as well. The three lattice degrees of proximity are put forward as a consequence:

$$ N\left( {S_{0}^{*} ,S_{1}^{*} } \right)\quad N\left( {S_{0}^{*} ,S_{2}^{*} } \right)\quad N\left( {S_{0}^{*} ,S_{3}^{*} } \right) $$

If there is $j_{0} \in \left\{ {1,2,3} \right\}$, the following formula (9) is put in play:

9 $$ N\left( {S_{0}^{*} ,S_{{j_{0} }}^{*} } \right) = \max \left\{ {N\left( {S_{0}^{*} ,S_{1}^{*} } \right),N\left( {S_{0}^{*} ,S_{2}^{*} } \right),N\left( {S_{0}^{*} ,S_{3}^{*} } \right)} \right\} $$

where upon based on the “the more moderate the better” rule in fuzzy algorithm, it could be confirmed that standard pattern *j*_0_ which the actual state of eco-tourism environmental carrying capacity of a given tourist destination is closest to and categorized into. Formula (9) is the evaluation matrix as for the actual state of eco-tourism environmental carrying capacity regarding a given tourist destination by means of fuzzy pattern recognition.

## An exemplary case study on the eco-tourism environmental carrying capacity of Kanas National Nature Reserves

Based on the eco-tourism environmental data of 2018 Kanas National Nature Reserves in Xinjiang, the eco-tourism environmental carrying capacity is exemplarily processed in this paper. Kanas National Nature Reserves are awarded to national 5A scenic area. Since the first tourist was received in 1997, the tourism in Kanas has been developing at a fast pace. With the implementation of Belt and Road Initiative strategy and the approval of the World Cultural Heritage application of “Silk Road” in 2022, the tourist attractions in Xinjiang along the Silk Road are once again warming up. Xinjiang accounts for six among the 33 representative sites successfully applied for World Heritage. The once dazzling pearl on the ancient Silk Road has again been glamorous to the global tourists. At present, Kanas National Nature Reserves, one of the two leading tourist destinations in Xinjiang, have been shaped as a quality national tourism route.

### Overview of the region

1. Nature resources

**Figure 4:**
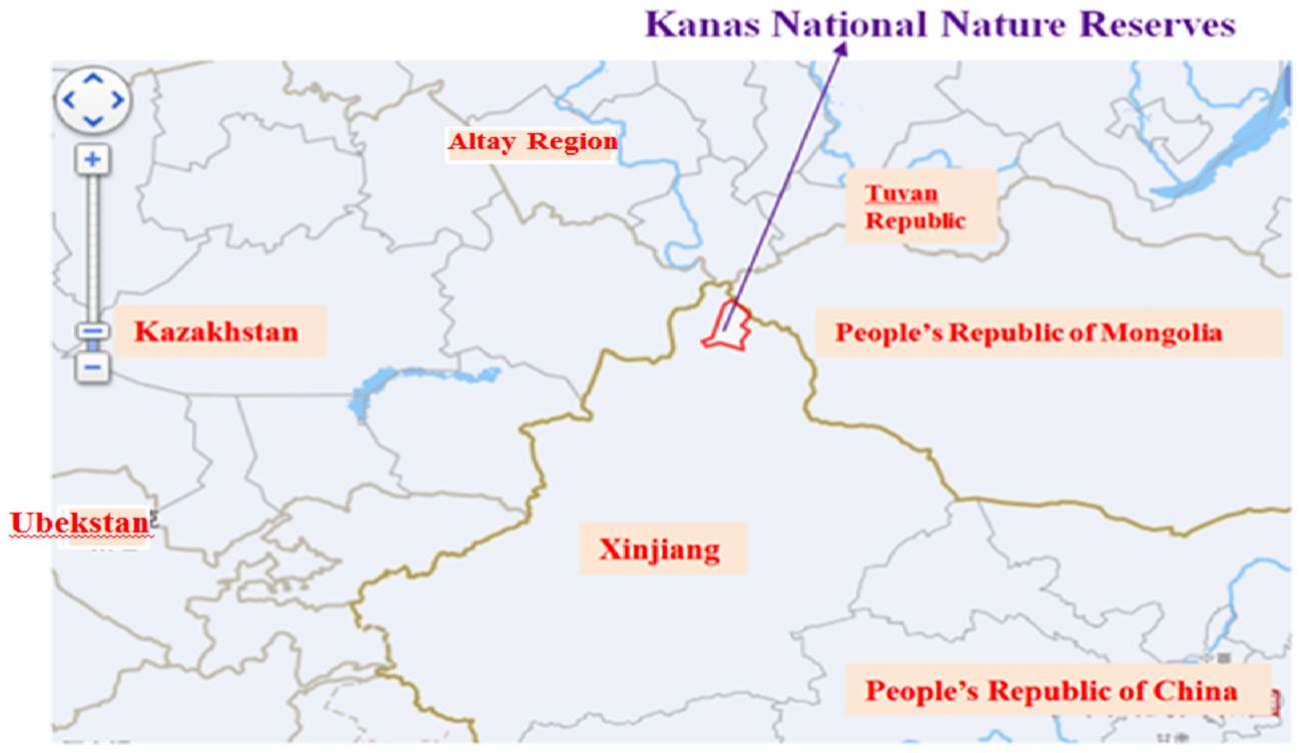
According to the Baidu Map, the area circled in red line is Kanas National Nature Reserves. It is located in northern Xinjiang, at the junction of China, People’s Republic of Mongolia, Kazakhstan and Tuva Republic.

Kanas National Nature Reserves are located at the junction of four countries (Fig. 4). Kanas tourist destination is centered on Kanas Lake which changes color with seasons and weathers. It is well-known as a “Color Changing Lake”, which is dyed with trees and surrounded by soaring snow peaks and bright mountains in every autumn. The scenery is picturesque.

Kanas tourist destination, located in the hinterland of Europe and Asia with high latitude, is featured with great changes in solar altitude angle with seasons, short time of direct exposure, heat disparity, which result in warm spring and autumn without summer climate characteristics, cold winter but not dramatic. The average annual temperature is − 0.2 °C, with the annual rainfall ranging from 780 to 1114 mm. Due to the complex terrain, the difference in vertical height, and the different climatic conditions related, various soil types have been developed in Kanas region. The complex naturally geographical environment in the area has brought rich animal and plant resources into being. At present, Kanas National Nature Reserves still maintains a primitive environmental state, providing a good place for the habitat and growth of wild animals and plants.

(2) Socio-cultural and economic conditions

The nature reserves have anciently been a nomadic area for many ethnic groups (i.e. Mongolian, Kazakh) from north, which are located in Kanas Village, Hemu Village, Baihaba Village, Tilekti village and Zibaroi, etc. Besides the tertiary industry related to tourism, the nature reserves are mainly in the line of animal husbandry and agricultural production business in small amount. In a word, the economic development level is still very low in the tourist destination.

### Eco-tourism in Kanas and Belt and Road initiative

Located in the hinterland of the Eurasian continent, Xinjiang is an important channel for the exchanges between Eastern and Western civilizations, an important junction on the ancient Silk Road, and a pivotal gateway for China to open to the west. Xinjiang is a pearl on the Belt and Road plate. Its unique geographical advantages and cultural spirits are full of mysterious colors, attracting tourists to explore here. The rapid development of transportation and infrastructure has remarkably propelled the tourism development in Xinjiang. On June 16, 2022, Alar Tarim Airport, the first geological airport built in the Tarim River basin, is opened up officially. The annual passenger throughput could be expected to reach 300 thousand person-time. In 2021, Xinjiang received 191 million tourists. As a name card of Xinjiang tourism, Kanas holds the position of enhancing the brand influence of Xinjiang tourism, establishing unique regional advantages, and promoting the development of tourism markets along the Belt and Road plate.

According to the “Classification, Investigation and Evaluation of Tourism Resources” (GB**/**T18972-2017) of the People’s Republic of China, the tourism resources in the region are classified and summarized in line with main category, sub-category, fundamental type of tourism resources and object of tourism resources. It shows that Kanas tourist destination is endowed with 8 main categories (geomatic landscape, water landscape, biological landscape, celestial and climatic landscape, architecture and facilities, historical relics, tourist purchases, humanistic activities) and 18 sub-categories which have been exploited to varying degrees. The main tourist attractions, in variety types of nature and culture, as well as rich landscapes, are concentrated in Kanas Lake, Hemu Village, Baihaba Village. In recent years, relying on the regional advantages in ecological environment, ice and snow attractions, folk culture, Kanas tourist destination has fully achieved a rapid development for being leisure, vacational village and health preservation. By virtue of the uniquely geographical advantages of Belt and Road Initiative, Kanas tourist destination prevails in becoming an area for international ski tourism.

With the Belt and Road Initiative, Kanas National Nature Reserves have seen a tourism boom in the past decade. Statistics show that the number of tourists to Kanas in 2018 was 22.83 million, more than seven times that of 2008. Tourism revenue was 23.4 billion RMB, 18 times that of 2008. On July 23, 2019, the number of tourists in Kanas scenic spots (Kanas core scenic spots, Baihaba scenic spots and Hemu scenic spots) exceeded 10 thousand a day, more than 20 days earlier than that in 2018^61^. The two peak tourist seasons in Kanas are generally during July to September in summer and the ski season from October to March of the following year. In recent years, with the entertaining and leisure facilities in Kanas getting further developed and improved, the peak tourist seasons are promisingly extended. At the same time, Kanas has also prompted its marketing and promotions, especially the hit of the variety entertainment program named “See You Again” in collaboration with a well-known Internet Video Platform in 2021.Kanas has become one of the tourist destinations that many young people most yearn for. This program, which was being filmed in Hemu scenic area and Kanas Lake scenic area, invited three celebrity couples with relationship problems to embark on an 18-day trip to Kanas. The audiences marvel at the magnificent and vast natural scenery as well as the exotic culture offered by Kanas, as a “fairyland on earth”, with the snow-capped peaks that never melt all the year round, the blue lake in the dense forest, and the arcadia-like villages. On July 9, 2022, even though the epidemic has not yet ended, with the aid of the local Corban Festival in Xinjiang, the average daily number of visitors to Kanas tourist attractions has surpassed 30 thousand, setting a new record for single-day tourist reception in recent years^62^. The 2023 Spring Festival marked the first tourism outbreak in China after the end of the epidemic in the past 3 years. On January 25, 2023, the three major ski resorts of Jungshan International Ski Resort in Altay City, Kokotohai International Ski Resort and Hemu (Jikepulin) International Ski Resort received a single-day high of 15 thousand tourists for sightseeing and skiing^63^.

### Environmental protection objectives for Kanas eco-tourism development

In compliance with the regulations pertinent to the classification and protection goals from the Ministry of Ecology and Environment of the People’s Republic of China, Kanas National Nature Reserves and Kanas tourist destination are functionally categorized into Grade I by the “Air Environmental Quality Guidelines” (GB3095-2012), while Kanas Lake is functionally classified into Grade I in line with the “Environmental Quality Guidelines for Surface Water” (GB 3838-2002).

### The computing process of Kanas eco-tourism environmental carrying capacity

In combination of general situations of eco-tourism development, as well as the uniquely natural, social, economic characteristics in Kanas tourist destination, the daily carrying capacity of spatial resource, ecological environment and economic environment in 2018 are obtained by applying related formulas and barrel theory. In the analysis of local history and reality, the daily carrying capacities of people’s psychological and socio-cultural environment are emerged^64^. Therefore, based on the calculation and analysis, the vectors *S*_1_**/***S*_2_**/***S*_3_ corresponding to the saturated**/**optimal**/**deficient standard patterns are determined. Then, the threshold vector *S*_max_ consequently comes out given by the following formula,

$$ {\text{Threshold}}\,{\text{Carrying}}\,{\text{Capacity}} = {\text{Suitable}}\,{\text{Carrying}}\,{\text{Capacity}} \times {12}0\%^{{{64}}} . $$

Since the consultation from the assessment group is not available so far, the threshold acquired by the above formula might be unreasonably less than the correspondingly numerical value regarding the saturated standard pattern. Hence, an appropriate amendment is made in this paper as follows,

$$ c_{{i{\text{max}}}} = c_{{i{1}}} \times {11}0\% $$

**Figure 5:**
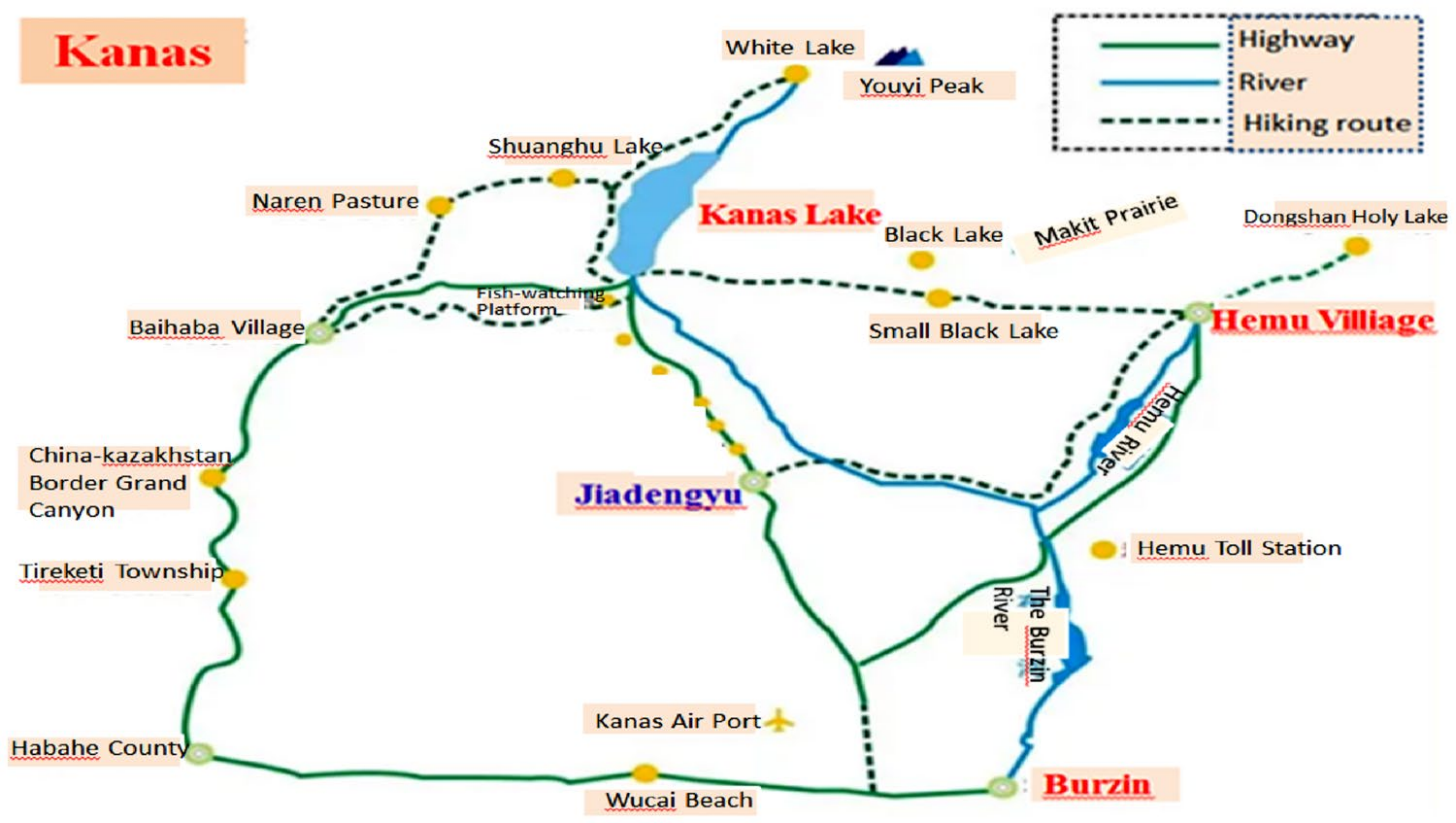
Touring routes of Kanas tourist attractions and main scenic spots (Kanas Lake, Hemu Villiage, Burzin, all marked in red) are displayed. Jiadengyu (marked in blue) is the entrance of the main scenic spots.

Kanas Lake is the main scenic spot of Kanas tourist attractions (Fig. 5) The touring routes to the Xiahukou Scenic Spot are verifiably pivotal to the threshold by calculating the spatial resource capacity of the Kanas Lake (Lake landscapes, Xiahukou Scenic Spot, Kanas Village). The daily carrying capacity of spatial resource is ascertained as follows,

$$ \begin{aligned} &c_{{{11}}} = {5789}\,{\text{person - time}}/{\text{day, }}c_{{{12}}} = {4728}\,{\text{person - time}}/{\text{day, }}c_{{{13}}} = {4}0{7}0\,{\text{person - time}}/{\text{day, }} \hfill \\ &c_{{{\text{1max}}}} = {6368}\,{\text{person - time}}/{\text{day}} \hfill \\ \end{aligned} $$

The ecological environment carrying capacity is constraining to the daily sewage treatment capacity. The maximum daily wastewater effluent of Kanas tourist destination is 590.50 m^3^**/**day. Sewage treatment capacity reaches 413.35 m^3^**/**day, given the sewage treatment rate as high as 70%, due to the continuous improvement of the drainage facilities^64^. The following can be deduced from this,

$$ c_{{{21}}} = {59}0.{5}0\,{\text{m}}^{{3}} /{\text{day, }}c_{{{22}}} = {413}.{35}\,{\text{m}}^{{3}} /{\text{day, }}c_{{{23}}} = {177}.{15}\;{\text{m}}^{{3}} /{\text{day, }}c_{{{\text{2max}}}} = {649}.{55}\,{\text{m}}^{{3}} /{\text{day}} $$

The daily accommodation capacity of tourist reception, composed of limited number of wooden homes and yurts outsourced by the individual enterprises, in addition to 3000 beds in different scale and grading, goes up to about 4000 beds^63^. The following data rises in response,

$$ c_{{{31}}} = {4}000\,{\text{beds, }}c_{{{32}}} = {32}00\,{\text{beds, }}c_{{{33}}} = {3}000\,{\text{beds, }}c_{{{\text{3max}}}} = {44}00\,{\text{beds}} $$

800 indigenous people and 1200 Hemu villagers, currently home in the main communities, are mostly nomadic people. The Tuva culture (i.e. the life style, religion, ritual activities) is the most distinctive folk custom featuring an atmosphere of antiquity and simplicity^64^. With the opening of family hotels and start-ups from the residents due to the rise of tourism, most of the Tuvas have being greatly improved their living conditions. The psychological carrying capacity of residents who are engaged in tourism as service or management personnel is conceivably close to infinite^64^. The 3-dimensional correlational vectors as following are generated by all accounts,

$$ S_{{1}} = ({5789,59}0.{5}0,{4}000) $$

$$ S_{{2}} = ({4728,413}.{35,32}00) $$

$$ S_{{3}} = ({4}0{7}0,{177}.{15,3}000) $$

$$ S_{{{\text{max}}}} = ({6368,649}.{55,44}00) $$

From 2011 to 2015, the number of tourists in Kanas has been massively increasing. In the peak season of 2018, the average number of arrivals soared to about 4000 person-time**/**day, and 6500 person-time**/**day for the rush hours^64^. Accordingly, the carrying capacity of spatial resource = (4000 + 6500) ÷ 2 = 5250 person-time**/**day; the sewage treatment rate = 80%, the carrying capacity of ecological environment = 472.4 m^3^**/**day; the carrying capacity of economic environment = 4000 beds; and people’s psychological capacity and socio-cultural capacity need not be discussed. Therefore, the actual state vector *S*_0_ in 2018 is,

$$ S_{0} = ({525}0,{472}.{4,4}000) $$

which is input into formula (6). The following membership vectors of the three standard patterns and the actual state membership vector are output in consequence,

$$ S_{{1}}^{*} = (0.{353}0,0.{25}00,0.{3333}) $$

$$ S_{{2}}^{*} = ({1,1,1}) $$

$$ S_{{3}}^{*} = (0.{5988,}0,0.{8333}) $$

$$ S_{0}^{*} = (0.{822}0,0.{7756,}0.{3333}) $$

According to formula (1), the lattice degrees of proximity between *S*_0_^*^ and *S*_1_^*^/*S*_2_^*^/*S*_3_^*^ is respectively calculated as follows,

$$ N\left( {S_{0}^{*} ,S_{1}^{*} } \right) = \frac{1}{2}\left[ {0.3530 + \left( {1 - 0.3333} \right)} \right] \doteq 0.5099 $$

$$ N\left( {S_{0}^{*} ,S_{2}^{*} } \right) = \frac{1}{2}\left[ {0.820 + \left( {1 - 1} \right)} \right] = 0.4100 $$

$$ N\left( {S_{0}^{*} ,S_{3}^{*} } \right) = \frac{1}{2}\left[ {0.5988 + \left( {1 - 0.7756} \right)} \right] = 0.4116 $$

In accordance with the evaluation matrix of formula (9), the indirect method of fuzzy pattern recognition is adopted to assess the actual state of eco-tourism environmental carrying capacity of Kanas tourist destination in 2018,

$$ N(S_{0}^{*} ,S_{{1}}^{*} ) = {\text{max}}\{ N(S_{0}^{*} ,S_{{1}}^{*} ),N(S_{0}^{*} ,S_{{2}}^{*} ),N(S_{0}^{*} ,S_{{3}}^{*} )\} $$

which illustrates that Kanas tourist destination is most approximate to or categorized into the saturated standard pattern, indicating that the tourism attractions are being overdeveloped right now in terms of the carrying capacities of spatial resource and ecological environment, which should be paid attention by the tourism monitoring authorities and operators.

## Discussion

### The applicability of fuzzy pattern recognition

Carrying capacity is not a formula of obtaining a fictitious number, beyond which development should cease, neither being fixed, but dynamic and resilient, which can’t go with an optimized answers or classification and be adaptive to the classic mathematics. Fuzzy sets can effectively describe the extension and intension of a concept. Pattern recognition refers to the science that concerns the description or classification (recognition) of measuring the noisy data and complex environment, followed by correct decisions. Carrying capacity can be manipulated by this managerial techniques and controls.

### The interpretation of the pattern recognition results

Generally speaking, the higher the numerical value of *N* (*S*_0_*, *S*_1_*) is, so is the higher degree of the overdevelopment. If it turns out the actual state of a given tourist destination is most approach to or categorized into the saturated/optimal/deficient standard pattern, the higher the numerical value of *N* (*S*_0_*, *S*_1_*) is, so is the higher degree of excessive/moderate/insufficient development. In particular, if it turns out that the actual state of the tourist destination is closest to or categorized into the saturated or insufficient standard pattern, each carrying capacity should be meticulously inspected. As a result, the actual state could be promisingly and accurately adjusted toward the optimal standard pattern.

### The reality of Kansas in corroboration with the discriminative results in the paper

In fact, with the incrementally convenient transportations, the development of tourist attractions and the improvement of tourism facilities, the arrivals to Kanas have been keeping at a high level in the past few years. It seems that the environmental carrying capacity symptomatizes being overloaded at the cost of compromising consumer experience. The so-called “three difficulties and one obstacle” (difficult to park, refuel, go to toilet; poor cell phone signal) is exactly reflecting the consumer complaint about the overcrowding of scenic spots. The environment is exposed to the high pressure from tourists, likewise. With the popularity of wild camping in China, more and more tourists become keen to camp in Kanas scenic spots wildly. Trampling on the grass and discarding garbage at will not only have an impact on the environment, but more seriously, an irreversible impact on the growth of rare tree species and the habitat of animals deriving from the deterioration of vegetation, water, landform and natural landscape.

## Conclusions

No remote islands or primitive tribes could be immune to the extremely developed transportation and networks nowadays. Eco-tourism could be unpredictably transformed into mass tourism by any chance, which must enormously make impact on the culture, style of life, and world-view of inhabitants of tourist regions. Hence, sustainable development is out of the question. This paper believes that the eco-tourism environmental carrying capacity, a most effective tool for the management department, of great importance to supervise the development state and distinguish which carrying capacity is about to be overloaded, and measures should decidedly be underway. Due to the carrying capacity being a dynamic and fluid concept, the fuzzy pattern recognition can be looked as categorization problem, as inductive process, as structure analysis, as discrimination method and so on concerning evaluating carrying capacity.

The executive steps are specifically as follows:

i. The measuring factors of each carrying capacity are ascertained by establishing the indicator of carrying capacity relevant to a given tourist destination.
ii. A decisively constraining measuring factor is figured out among the measuring factors of each carrying capacity by the assessment group organized by the development investors, tourism management department, local government, experienced experts and local residents. The numerical values of the three standard patterns and standard pattern matrix, as well as the vectors of threshold and the actual state are confirmed in sequence by the help of field investigation.
iii. The vectors of the three standard patterns and the actual state are processed in normalization by implementing “the more moderate the better” rule, as a consequence, the corresponding membership vectors and the membership matrix of the standard patterns are got hold of.
iv. In comparison of the lattice degrees of proximity, the actual state could be identifiably proximal to which standard (saturated**/**optimal**/**deficient) pattern of development. The result plays a role in being a reference to the decisions on the next step development.
v. The case of Kanas clarifies that tourism had been pro to destruction of the natural elements that form the basis of the tourist products. Negative effects are aligned with motion, staying, and different forms of leisure activities leading to pollution and quantitative decrease of natural resources, to the endangerment of the unmolested and diverse wildlife, and in many places to the destruction of natural landscapes, which should sound an alarm across the actors with the tourism management department.

In recent years, the tourism carrying capacity is entitled to the wisdom featured tourism research. The tourism carrying capacity state of the protected areas could be synchronously measured by means of the advanced information technology for establishing an early warning mechanism, such as the use of location information to collect tourist flow and tourism behavior data. On the other hand, dynamic surveillance and early warning could be realized in a manner of digital monitoring. For example, dynamic data of tourist flow and spatial distribution could be grasped in combination of various sensors of each scenic spot and tourist data management system for monitoring regional carrying capacity. In addition, more and more methods (i.e. system dynamics model) are used to measure the growth limit and ecological vulnerability of tourist attraction and to estimate the potential risks related, for the purpose of timely adjusting strategies and maintaining the sustainable development of local tourism.

## Data Availability

All data generated or analyzed during this study are included in this published article.
